# Annotations

**Published:** 1892-04-30

**Authors:** 


### Annotations.

**The Clergy and Vivisection.**

The next Church Congress will be held at Folkestone, on October 4th, and Bishop Barry is announced to speak on the subject of vivisection. The resolution to be submitted is: "Do the interests of mankind require experiments on living animals, and how far are they justifiable?" It is right and wise that the clergy, as public instructors in morals, should carefully consider such a comparatively novel subject as vivisection, from the standpoint of an absolute morality. Perhaps the clergy themselves will admit that they are not quite competent to decide by themselves the question whether or not the interests of mankind require experiments on living animals. At least, they will grant that they are not proper persons to determine the quality and amount of definite physiological knowledge which experiments on living animals have gained for science. In medicine, we are apt to consider that the prime requisite for a public speaker is that he should know at least a great deal about the subject which he undertakes to discuss. Bishop Barry will, we think, agree with us that this view of things is sound in principle. We would suggest to him, therefore, that he and half-a-dozen other intelligent clergymen should pay a series of visits to some physiological laboratory, observe for themselves, without passion, the methods of physiological experimentation there employed, and discuss with trusted physiological experimenters the reasons for and against their work. It is absurd that such a subject should be treated by educated persons like clergymen and doctors with heat and passion on both or on either side. Doctors, at least the cultured and scientific ones, have as just an appreciation of the value of a high standard of public morals as the clergy have. The clergy can discuss the moral aspect of the question alone with anything like profit. If they wish to discuss even that question in a reasonable and Christian spirit they must call to their aid a number of trusted members of the medical profession who acknowledge obligations to vivisection. Passionate shoutings and stampings of the feet may be appropriate enough for electioneering meetings, but when methods of scientific discovery and progress are at stake it is obvious that right reason alone should be called upon to decide.

**Mr. Goschen and Doctors' Incomes.**

The Chancellor of the Exchequer is an unlucky kind of man. This year, without intending it, he has been unfortunate enough to annoy the members of the medical profession by his Budget references. He institutes a comparison between doctors and cotton spinners, and makes it appear that the former are more prosperous than the latter, because, taken collectively, they make a better show in his Income-tax returns. He might just as well institute a comparison between the Bench of Bishops and the Chancellor of the Exchequer. There can be no true comparison between these classes, because there are nearly thirty bishops but only one Chancellor of the Exchequer. Similarly there are about thirty thousand doctors, but not, perhaps, more than two or three thousand cotton spinners, great and small. As doctors are a more expensively educated class than cotton spinners, it ought not to greatly surprise even a Chancellor of the Exchequer that ten of the former should unitedly earn a larger income than one of the latter. Perhaps Mr. Goschen really believes that one cotton spinner is entitled to as large an income as ten doctors. But much as we appreciate cotton-spinners, we cannot quite endorse an opinion of that kind. We have often pointed out in these pages that a large proportion of medical men cannot earn a living at all, do as they will; that another large proportion earn only a bare subsistence by an amount of strenuous and persistent toil which abridges the average duration of their lives by seven to twelve years; that a very small proportion attain to an exceedingly modest competency at an advanced age; and that perhaps one doctor in every ten years dies, leaving a fortune behind him. This, we believe to be a just presentation of the financial aspect of the medical profession. If money be the chief object of work and desire in the present day, as it undoubtedly is, then the medical profession can show a larger proportion of disappointed persons than any other calling, unless, perhaps, we except the clerical. Persons who have even a faint regard for money should shun medicine as they would like to shun that myrmidon of the Chancellor of the Exchequer—an income tax collector.

**Ladies' Chins.**

The modern British artist, caught by the specialisation craze, seems to be concentrating his attention on ladies' chins. This is especially the case with the fashion-plate artist. Human chins may be divided into four classes. First, the retreating chin, which falls away behind the frontal line of the face. This is the pet abomination of modern chin-fanciers. Secondly, the normal chin, which is a chin of moderate size, definite outline, and flush with the frontal line of the face; this ought to be the chin approved by painters. Thirdly, there is the long prominent chin, which is pushed forward somewhat in advance of the frontal line of the face; this is ugly, though not always markedly so. Fourthly, there is the very long, very large, and decidedly protruding chin; this, in scientific language, is the prognathous chin, the chin, in fact, of the monkey tribe. It is the chin, slightly modified, which the highly cultured artists of the fashion-plates have chosen as the type of the beautiful in female chins. Darwin had something to say on the point. In the "Descent of Man" he wrote: "The early male forefathers of man were probably furnished with great canine teeth, but as they gradually acquired the habit of using stones, clubs, or other weapons for fighting with their enemies or rivals, they would use their jaws and teeth less and less. In this case, the jaws, together with the teeth, would become reduced in size." What strikes one here is that large jaws and a prominent chin in man appear to have been needed, at a low stage of development, for fighting with the mouth and teeth as monkeys and dogs fight. If the fashion-plate artists of to-day are showing us real and not imaginary types of female beauty, it would seem that women are beginning to revert to a condition of low development similar to that occupied by man in his transition from the simian to the bimanous stage. If this be so we may anticipate, since Nature seldom does anything without a purpose, that the quarrels of women, more especially of fashionable women, will soon begin to be fought out, not in the law courts or by polite letter writing and the social "cut," but by the good old method of "tooth and nail." We commend these developmental facts to the consideration of the admirers of huge and hideous chins.

